# Key Players in Choline Metabolic Reprograming in Triple-Negative Breast Cancer

**DOI:** 10.3389/fonc.2016.00205

**Published:** 2016-09-30

**Authors:** Egidio Iorio, Maria José Caramujo, Serena Cecchetti, Francesca Spadaro, Giulia Carpinelli, Rossella Canese, Franca Podo

**Affiliations:** ^1^Department of Cell Biology and Neurosciences, Istituto Superiore di Sanità, Rome, Italy; ^2^Department of Hematology, Oncology and Molecular Medicine, Istituto Superiore di Sanità, Rome, Italy

**Keywords:** triple-negative breast cancer, phosphatidylcholine metabolism, metabolic reprograming, phospholipase, choline kinase

## Abstract

Triple-negative breast cancer (TNBC), defined as lack of estrogen and progesterone receptors in the absence of protein overexpression/gene amplification of human epidermal growth factor receptor 2, is still a clinical challenge despite progress in breast cancer care. ^1^H magnetic resonance spectroscopy allows identification and non-invasive monitoring of TNBC metabolic aberrations and elucidation of some key mechanisms underlying tumor progression. Thus, it has the potential to improve *in vivo* diagnosis and follow-up and also to identify new targets for treatment. Several studies have shown an altered phosphatidylcholine (PtdCho) metabolism in TNBCs, both in patients and in experimental models. Upregulation of choline kinase-alpha, an enzyme of the Kennedy pathway that phosphorylates free choline (Cho) to phosphocholine (PCho), is a major contributor to the increased PCho content detected in TNBCs. Phospholipase-mediated PtdCho headgroup hydrolysis also contributes to the build-up of a PCho pool in TNBC cells. The oncogene-driven PtdCho cycle appears to be fine tuned in TNBC cells in at least three ways: by modulating the choline import, by regulating the activity or expression of specific metabolic enzymes, and by contributing to the rewiring of the entire metabolic network. Thus, only by thoroughly dissecting these mechanisms, it will be possible to effectively translate this basic knowledge into further development and implementation of Cho-based imaging techniques and novel classes of therapeutics.

## Introduction

**Figure 1:**
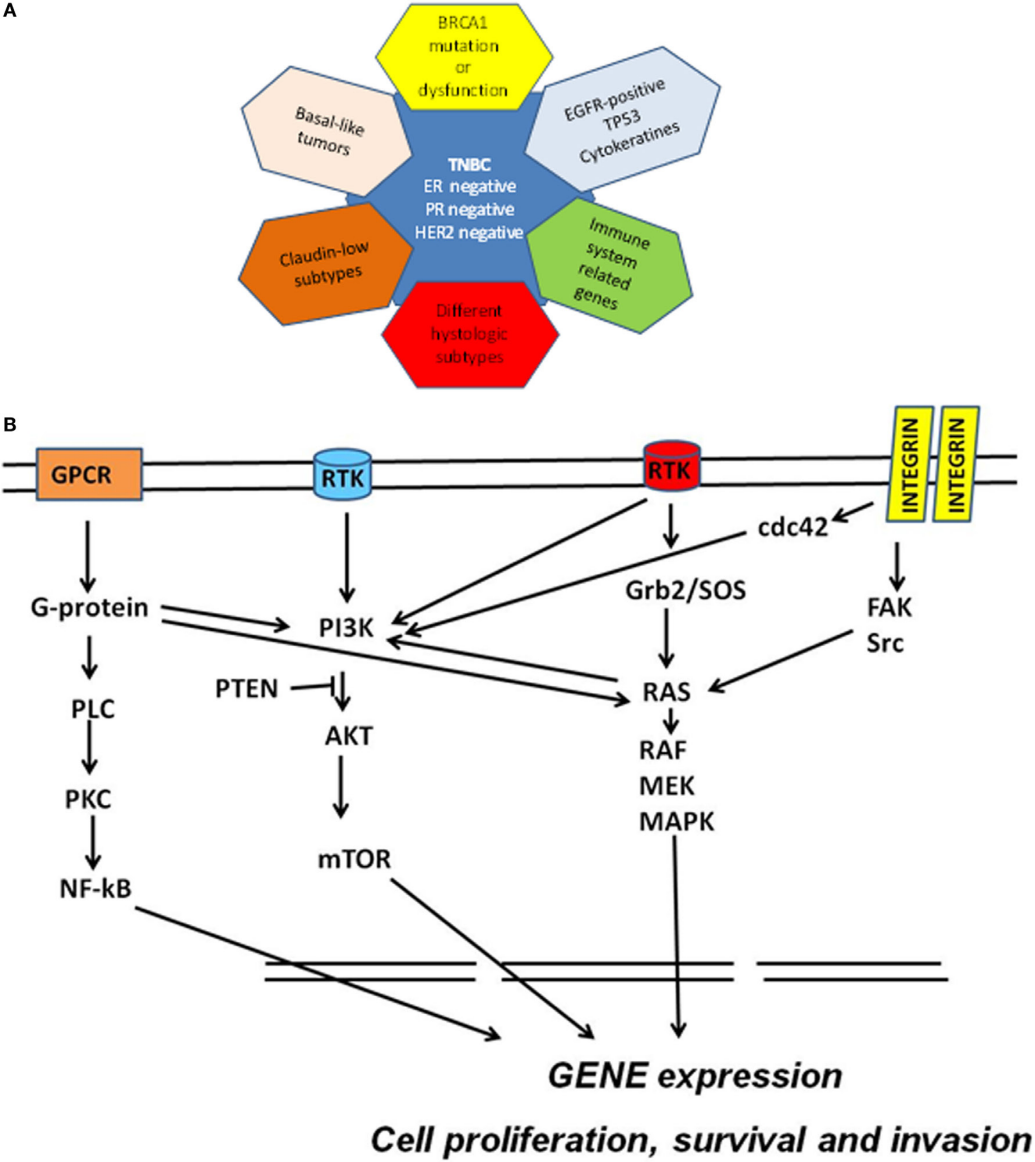
**(A)** Molecular subclassification of triple-negative breast cancer (TNBC) based on gene expression profiling. Triple-negative breast cancers have been defined as tumors that are devoid of the expression of estrogen receptor (ER), progesterone receptor (PR), and human epidermal growth factor receptor 2 (HER2). TNBC overlap with (1) basal-like breast cancers; (2) BRCA-mutated tumors; (3) claudin-low tumors; (4) tumors overexpressing EGFR, associated with TP53 mutations or expressing cytokeratins; (5) tumors characterized by immune response signatures; and (6) tumors possessing some special histological types. **(B)** Schematic molecular pathway identified in TNBC. Multiple signaling cascades are activated in TNBC including those triggered by receptor tyrosine kinases (RTK), G protein-coupled receptor (GPCR), and integrins and their downstream effectors. Ras-mediated signaling commonly occurs through the RTK/growth factor receptor-bound protein 2 (Grb2)/Sos–Ras pathway. Ras directly interacts with and activates Raf. Raf phosphorylates and activates MEK, which in turn phosphorylates and activates MAPKs. Integrin engagement triggers several signaling cascades including those that are mediated by FAK, Src, and cdc42. Activation of RTK and other external stimuli lead to the activation of PI3K pathway. PI3K activates AKT (whereas PTEN inhibits this activation) and then mTOR. The G-proteins bind and activate phospholipase C and activate the nuclear factor kappa B (NF-κB) transcription factor. This network of cell signaling pathways result in the activation of transcription factors that drive genomic signature programs of dysregulated cell cycle progression, proliferation, invasion, and survival.

Classification of breast cancer (BC) has been historically based on both analysis of tumor morphology and histological detection of three marker proteins: the estrogen receptor (ER), the progesterone receptor (PR), and the human epidermal growth factor (EGF) receptor tyrosine kinase 2 (ErbB2 or HER2). Tumors which express none of these three markers are collectively referred to as triple-negative breast cancer (TNBC; ER^−^, PR^−^, HER2^−^) and still pose a clinical challenge. More recently, gene expression analyses showed that BC is a more heterogeneous disease than previously assumed and the BC histotypes based on ER/PR/HER2 classification were expanded to include five major transcriptional subtypes: basal-like, HER2-enriched, luminal A, luminal B, and normal breast-like (1, 2). The majority (70–80%) of TNBCs are defined as basal-like by gene expression (3) and share other molecular features with this BC subtype. According to most epidemiological studies, TNBCs represent 10–20% of all BCs, although a higher proportion can be found in some ethnic groups and among BRCA1 mutation carriers. TNBCs are typically poorly differentiated, frequently have high histological grade and mitotic index, and often present early onset and shorter disease-free and overall survival (4, 5). Recent large-scale gene expression and genome-based studies have shown that TNBC is a heterogeneous disease (Figure 1A) comprising at least four to six definable molecular subtypes that express elements of distinct oncogenic signaling pathways (6, 7). Interestingly, 50–70% of all TNBCs overexpress the EGF receptor tyrosine kinase 1 (ErbB1, EGFR, or HER1). EGFR is one of the major regulators of survival, proliferation, and migration (8), leading to activation of the phosphoinositide 3-kinase (PI3K) and ERK pathways, which in turn stimulate other receptor-activated signaling cascades associated with cancer onset and progression (Figure 1B) (9).

The aggressiveness of TNBC and the lack of targeted therapies specifically recommended for TNBC patients highlight the need to explore additional molecular mechanisms beyond genomic and proteomic changes to better elucidate the metabolic pathways required for TNBC growth and survival (10, 11).

## Metabolic Reprograming in TNBC Cells

Molecular genomic and proteomic studies have been carried out to understand the complexity of TNBC and identify markers that can be therapeutically targeted. However, little is known about the metabolic alterations that distinguish TNBC from non-triple-negative subtypes and characterize TNBC progression. Previous studies have shown that proteins that are involved in glycolysis, glutaminolysis, and glycine or serine metabolism are differentially expressed among different BC subtypes in tissue microarray sections and in a large series of invasive BC specimens (12–14). In particular, several observations reported that TNBC have elevated glucose uptake and a glycolytic gene/protein expression signature (12, 14). A recent study reported a novel mechanism, whereby the transcription factor c-Myc drives glucose metabolism in TNBC MDA-MB-157 cells by direct repression of thioredoxin-interacting protein (TXNIP), a potent negative regulator of glucose uptake, aerobic glycolysis, and glycolytic gene expression. A Myc^high^/TXNIP^low^ gene signature correlates with decreased overall patient survival and decreased metastasis-free survival in BC. The correlation between the Myc^high^/TXNIP^low^ gene signature and poor clinical outcome is evident only in TNBC, not in other BC subclasses. Furthermore, mutation in p53 (TP53), found in the majority of TNBCs, enhances the correlation between the Myc^high^/TXNIP^low^ gene signature and death from BC (15). Finally, an increase of glycolysis was found in a panel of five TNBC cells and accumulation of fructose-1,6-bisphosphate (F1,6BP), a glycolytic intermediate that directly binds to and enhances the activity of EGFR, was detected in MDA-MB-468 cells, with enhanced lactate excretion, tumor growth, and immune escape (16). TNBC cell lines, such as MDA-MB-468, MDA-MB-231, MDA-MB-436, and BT20, exhibit function defects in multiple respiratory complexes with a reduction in expression of complex I and complex III proteins of the mitochondrial respiratory chain relative to receptor-positive cell lines (17). The increased glycolytic activity in TNBCs could be responsible for the increased ^18^F-fluorodeoxyglucose (FDG) uptake generally reported in PET examinations of these patients, beyond a large variability in the maximum and mean standardized uptake values and in metabolic volumes (18–21).

Lipid metabolism activation in BC cells is recognized as a hallmark of carcinogenesis (22, 23). Increased fatty acid (FA) synthesis due to increased levels of fatty acid synthase (FAS) has been observed in various cancers and is correlated with a poor prognosis in many instances (23). FAS could, in principle, be an appealing therapeutic target because most cancer cells depend on FAS-mediated *de novo* FA synthesis, whereas most healthy cells prefer to incorporate exogenous FAs (24). However, reports on the overexpression of FAS across BC subtypes, and in TNBC in particular, are still contradictory (25–27), and further studies are needed before considering this enzyme as a strong therapeutic target.

The concerted activation of an assembly of molecular complexes in cancer cells cooperates to sustain an oncogene-induced cell signaling through multiple postreceptor pathways involved in phospholipid biosynthesis and breakdown. Among these, phosphatidylinositol 4-phosphate 5-kinase Igamma (PIPKIγ) is overexpressed in TNBC cells, in which the loss of this enzyme impairs PI3K/Akt activation (28). Furthermore, two major enzymes involved in the agonist-induced phosphatidylcholine (PtdCho) cycle, such as choline kinase (ChoK) and PtdCho-specific phospholipase C (PC-PLC), are overexpressed and activated in various BC subtypes, including TNBC cells, with the implications on expression and oncogenic function of EGF receptors’ family members (29–33).

**Figure 2:**
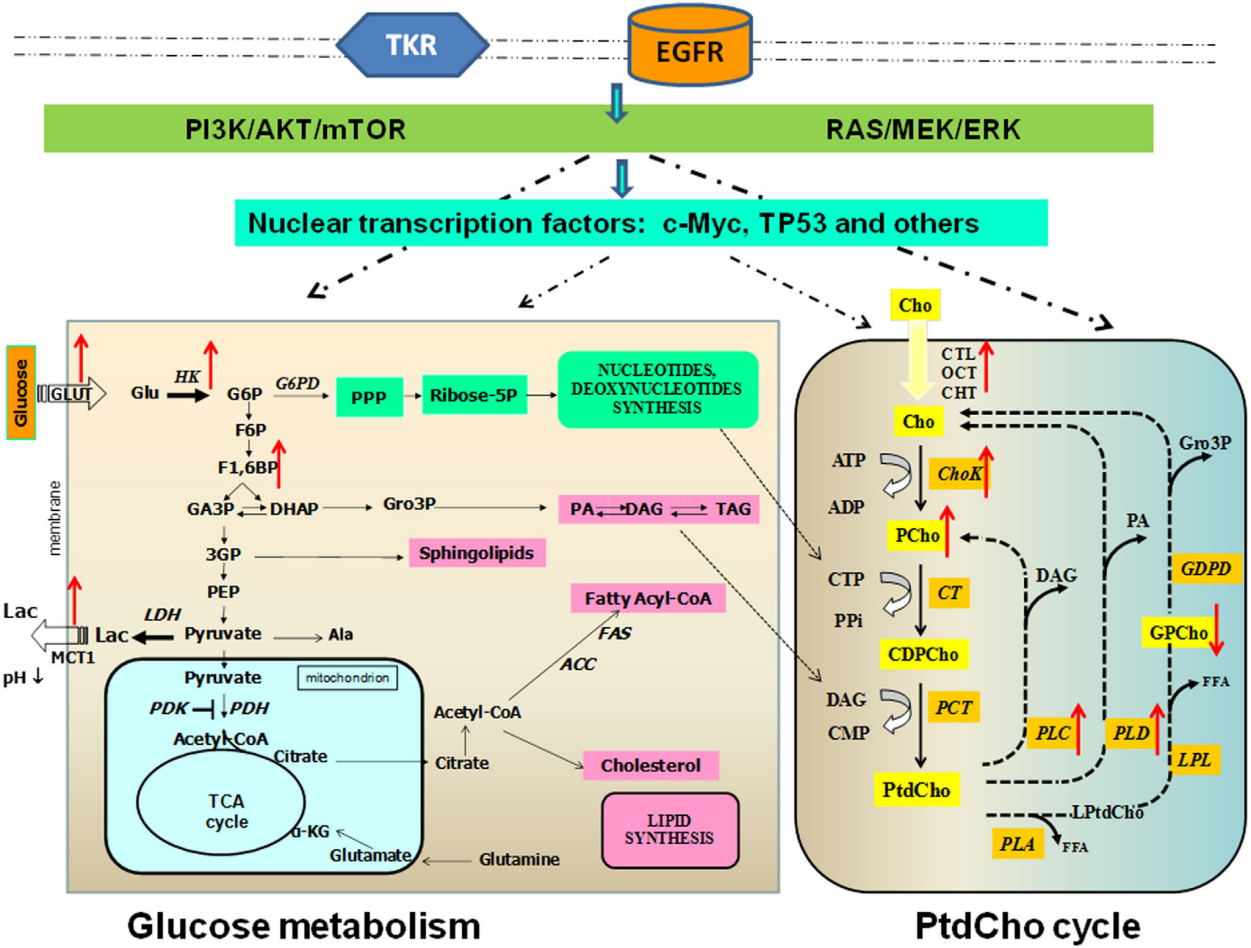
**Links between altered glucose and phosphatidylcholine metabolism in breast cancer**. Glucose metabolism occurs in cancer cells. Glycolysis is a series of metabolic processes, in which 1 mol of glucose is catabolized to 2 mol of pyruvate. As indicated, several intermediates can fuel the pentose phosphate pathway (PPP) or lead to lipid synthesis. In cancer cells, pyruvate is further converted into lactate. Pyruvate can be imported in the mitochondrial matrix to feed the tricarboxylic acid (TCA) cycle. This step is controlled by pyruvate dehydrogenase kinase (PDK), which can inactivate pyruvate dehydrogenase (PDH). *Transporters*: Glut, glucose transporter; MCT, monocarboxylate transporter. *Metabolites*: Ala, alanine; α-KG, α-ketoglutarate; DAG, diacylglycerol; G6P, glucose-6-phosphate; F6P, fructose-6-phosphate; F1,6BP, fructose-1,6-bisphosphate; DHAP, dihydroxyacetone phosphate; Gro3P, sn-glycerol-3-phosphate; GA3P, glyceraldehyde-3-phosphate; G3P, 3-phosphoglycerate; PA, phosphatidate; PEP, phosphoenolpyruvate; TAG, triacylglycerol. *Enzymes*: ACC, acetyl-CoA carboxylase; FAS, fatty acid synthase; HK, hexokinase; LDH, lactate dehydrogenase; PDK, pyruvate dehydrogenase kinase; PDH, pyruvate dehydrogenase phosphatidylcholine (PtdCho) cycle. *Transporters*: CHT1, choline high-affinity transporter-1; CTL, choline transporter-like protein; OCT2, organic cation transporter-2. *Metabolites*: CDP-Cho, cytidine diphosphate choline; Cho, free choline; DAG, diacylglycerol; FFA, free fatty acid; Gro3P, sn-glycerol-3-phosphate; GPCho, glycerophosphocholine; LPtdCho, lysophosphatidylcholine; PA, phosphatidate; PCho, phosphocholine. *Enzymes*: Kennedy pathway: ChoK, choline kinase (EC 2.7.1.32); CT, cytidylyltransferase (EC 2.7.7.15); PCT, phosphocholine transferase (EC 2.7.8.2). Headgroup hydrolysis pathways: PLC, phospholipase C (EC 3.1.4.3); PLD, phospholipase D (EC 3.1.4.4). Deacylation pathway: PLA1, phospholipase A1 (EC 3.1.1.32); PLA2, phospholipase A2 (EC 3.1.1.4); LPL, lysophospholipase (EC 3.1.1.5); PD, glycerophosphocholine phosphodiesterase (EC 3.1.4.2). Red arrows indicate direction of change in enzyme activity enzymes and metabolite content.

The present evidence points to the existence of multiple links between enzymes involved in the glycolytic gene/protein signature and those responsible for enhanced carbon fluxes through the oncogene-driven PtdCho biosynthesis and catabolism in BC cells (Figure 2). This biochemical interplay may also serve as a key regulator of tumor progression in TNBCs.

## PtdCho Metabolism in TNBC

The introduction of magnetic resonance spectroscopy (MRS) in cancer biology allowed the detection of abnormal profiles of aqueous total choline-containing metabolites (tCho) of the PtdCho cycle in cancer cells and tissues, both at preclinical and clinical level (9, 34–37). Substantially modified ^1^H MRS tCho spectral profiles have been reported on malignant transformation of human mammary (38–40) and prostate epithelial cells (41) and in ovarian cancers (42–44). These modifications occur in the 3.20–3.24 ppm ^1^H MRS spectral region and are typical of the trimethylammonium headgroups of PtdCho precursors and catabolites, such as phosphocholine (PCho), glycerophosphocholine (GPCho), and free choline (Cho).

Phospholipids play the dual role of being basic structural components of membranes and acting as substrates of reactions involved in key regulatory functions in mammalian cells (45, 46). Hydrolysis of PtdCho, the most abundant phospholipid in eukaryotic cell membranes, can generate second messengers, such as diacylglycerol (DAG), phosphatidic acid (PA), lysophosphatidic acid (LPA), arachidonic acid (AA), and lysophosphatidylcholine (LPtdCho). These PtdCho metabolites are produced through three major catabolic pathways, respectively, mediated by specific phospholipases of type C (PC-PLC) and D (PLD), which act at the two distinct phosphodiester bonds of the PtdCho headgroup, and by phospholipases of type A2 and A1 (PLA2 and PLA1), which act in the deacylation reaction cascade (Figure 2). PCho accumulation either produced by ChoK in the first reaction of the three-step Kennedy biosynthetic pathway or by PLC-mediated PtdCho catabolism is associated with tumor growth and progression (9, 33–36).

This accumulated evidence supports the inclusion of an altered phospholipid metabolism as a novel candidate hallmark for cancer and as a key regulator in the overall cancer metabolic reprograming. An aberrant PtdCho metabolism associated with increases in the intracellular total choline-containing PtdCho metabolites (tCho) and phosphocholine (PCho) contents (femtomoles per cubic micrometer cell) were initially observed in BC cells as they progressed from normal to malignant phenotypes, i.e., from non-tumoral immortalized MCF-12A to the highly metastatic TNBC MDA-MB-231 and MDA-MB-435 cell lines (38). The high intracellular content of PCho seemed unrelated to the demand for cell membrane biosynthesis in the investigated BC cells characterized by different doubling time values (38). Notably, a different rate of increase was observed for PCho (6×) and PtdCho (1.5×) in MDA-MB-231 BC cells compared with the non-malignant MCF12A cells (40). An integration of MRS with gene microarray analysis revealed that a combination of upregulated ChoK and PLD and/or an increased PC-PLC expression/activity caused PCho accumulation in MDA-MB-231 cells, while lower levels of GPCho were consistent with underexpression of cytosolic calcium-dependent PLA2 group IVA and lysophospholipase 1 (40). Elevated levels of ChoKα and PLD1 isoforms were found in both the ER^−^ MDA-MB-231 BC cell line and in patient-derived ER^−^ BC specimens, as compared with the corresponding non-metastatic ER^+^ MCF-7 BC cell line and ER^+^ patient-derived BC samples (47). Downregulation of ChoKα by RNA silencing increased PLD1 expression, and downregulation of PLD1 increased ChoKα expression, indicating a close relationship between ChoK and PLD enzymes (47). Additionally, ChoKα silencing resulted in increased PC-PLC protein expression (e.g., twofold in MDA-MB-231) suggesting that BC cells could compensate for the loss of ChoKα protein levels with PC-PLC upregulation, thus maintaining an intracellular PCho pool size markedly higher than that of non-tumoral breast epithelial cells (48, 49).

Although the role of PLAs in BC cells remains unknown, very low expression of secreted PLA2 (sPLA2) was found in basal-like breast tumor biopsies and cultured cells (50). As noted by the authors, the mRNA expression of sPLA2s belonging to IIA, III, and X groups is regulated by DNA methylation and histone deacetylation, and all three genes are significantly silenced in aggressive TNBC cells due to both mechanisms. It may be interesting to investigate a possible relationship with the regulation of cytosolic PLA2s underexpressed in TNBC apparently linked to low levels of GPCho in this BC subtype (40).

A study by Eliyahu and colleagues (39) confirmed an altered PtdCho metabolism in different molecular BC subtypes relative to human mammary epithelial cells (HMECs), although the MDA-MB-231 TNBC cells exhibited the lowest PCho/NTP ratio among the investigated BC cell lines. Interestingly, under the adopted experimental conditions, the PCho level in HMEC and BC cells was found to correlate with Cho transport into the cells, mainly due to the organic cation transporter-2 (OCT2) and the choline high-affinity transporter-1 (CHT1), but not with ChoK activity, suggesting that this step is fast and not rate limiting, although its induction ensures increased PCho levels. The upregulation of choline transporters and ChoK may be related to a cascade of genetic changes that are associated with the multistep process of carcinogenesis (51).

Enzymatic assays showed a twofold to sixfold activation of PC-PLC in BC of different subtypes compared with a non-tumoral counterpart (MCF-10A cell line) (32). The activity rate measured in the TNBC MDA-MB-231 cell line was about twofold higher than that of HER2-enriched and ER-positive cell lines. Metabolic analysis of MDA-MB-231 cells identified a characteristic biochemical signature of these cells relative to the non-tumoral MCF-10A counterpart, consisting of higher contents of PCho and succinate, elevated proportion of monounsaturated FAs and increased ChoK and PC-PLC protein expression (52). The importance of the cell membrane lipid profile to discriminate BC subtypes is receiving increasing attention, and the results suggest possible links between altered metabolic pathways in BC and membrane molecular rearrangement. He and colleagues (53) could discriminate BC cell lines from MCF-10A cells on the basis of phospholipid species composition and expression of five lipogenesis-related enzymes. The authors suggested that elevated expression levels of fatty acid synthase 1 (FAS1), stearoyl-CoA desaturases 1 and 5 (SCD1 and SCD5), and ChoKα may be closely related to enhanced levels of saturated and monounsaturated lipids in BC cell lines. Most interestingly, it was possible to distinguish adequately between MDA-MB-231 cells based on the highest level of PtdCho (36:1) and PtdCho (36:2) and ChoKα expression compared with other BC subtypes (53).

Differences in phospholipid and lipid metabolism between cells in culture and in solid tumors have been detected by *in vivo* MRS in the MDA-MB-231 model. These differences may be ascribed to characteristic conditions of solid tumor microenvironment such as depletion of nutrients and oxygen, changes in pH, and interactions between cancer and stromal/endothelial cells (54, 55). A higher concentration of tCho (mainly due to PCho) was found in hypoxic regions of heterogeneous orthotopic MDA-MB-231 tumor xenografts using three-dimensional multimodal molecular imaging platforms (56, 57). The high tCho content of tumor cells was associated with enriched levels of proteins involved in glucose metabolism, PI3K-Akt/Ras/FAS signaling pathway, protein processing in endoplasmic reticulum, apoptosis, and telomere stress-induced senescence (56). A stable silencing of glycerophosphodiester phosphodiesterase domain containing 5 (GDPD5), which is upregulated in TNBC cells and tumors, induced an increase in the levels of GPCho and phosphoethanolamine in MDA-MB-231 BC cells and in their orthotopic tumor xenografts compared with controls (58, 59) suggesting a close networking between choline and ethanolamine phospholipid cycles. A larger variation in the PCho/GPCho ratio was observed for the basal-like BC subtype of patient-derived xenograft models relative to luminal B subtype xenografts (60, 61). These differences could be explained by lower mRNA expression of ChoK (α and β) and higher expression of PLA group 4A in basal tumor xenografts (61). In another experimental subcutaneous model expressing basaloid TNBC markers (HCC1806), phosphomonoesters (which include PCho) and lactate levels were modulated by tumor size (62).

The metabolic characterization of TNBC human biopsies is far from being well established, although different studies reported a higher overall tCho content in TBNCs with respect to non-tumoral tissues. A significantly higher tCho level, either quantified in millimoles per kilogram or expressed as peak integral normalized to the volume of interest and the signal-to-noise ratio, was reported in TNBC compared with non-triple-negative tumors using *in vivo* MRS (63, 64). A quantitative study on *in vivo* MRS examinations of 29 patients indicated that TNBCs exhibited increased tCho levels ranging from 0.4 to 4.9 mmol/kg (65). TNBCs are more likely to have high tCho than other BC subtypes, although the situation may be reversed in young age patients (range 28–39 years) (66). The high tCho levels were well correlated with the standardized FDG uptake value obtained using PET/CT and with the histological prognostic parameters (19). A recent paper (67) reported that intact TNBC biopsies could be discriminated by HR-MAS analyses from triple-positive BCs based on their content in free Cho and GPCho, which were significantly higher in TNBC relative to triple-positive BC. In this paper (67), the analyzed TNBC biopsies contained lower levels of glutamine and higher levels of glutamate compared with tumors with positive receptor statuses which might result from increased glutaminolysis metabolism and suggests dependence on glutamine to support cell growth. This issue warrants measurements of metabolic fluxes using suitable isotopomeric models, such as bonded cumomer analysis by MRS or fragmented cumomer analysis by mass spectrometry (68, 69).

## Targeting PtdCho Metabolism in TNBC

The role of PtdCho cycle enzymes as potential new molecular targets in TNBC can be investigated using molecular depletion approaches and/or pharmacological inhibitors. However, there are no studies that allowed discrimination of specific effects of targeting PtdCho-cycle enzymes on TNBC versus other BC subtypes, and most of studies were performed using only one or two TNBC cell lines. Although a reduction of 85% of *in vivo* tumor growth was obtained in subcutaneous MDA-MB-231 tumors treated with the ChoK inhibitor Mn58b (70), this result needs further evaluation in different TNBC models. The downregulation of ChoK in MDA-MB-231 cells cultured *in vitro* induced profound alterations in cell proliferation and promoted differentiation, as detected by cytosolic lipid droplet formation and modified expression of galectin-3 (71). These changes were associated with alterations of 33 proliferation-related genes and 9 DNA repair-related genes (72). Interestingly, in MDA-MB-231cells, a combination of ChoK silencing with a conventional treatment using 5-fluorouracil resulted in higher cell death rate relative to that obtained when each treatment was applied individually (72) confirming a key role for the ChoK enzyme in *in vitro* cell proliferation and survival. Furthermore, *in vivo* targeting of ChoK by either lentiviral gene silencing (73) or by ChoKα depletion using specific short-hairpin RNA (shRNA) (74), respectively, induced a growth delay or strongly repressed tumor growth in MDA-MB-231 xenograft bearing mice.

Additionally, there is mounting evidence from studies on experimental TNBC models that the reduction/destabilization of ChoK protein levels rather than inhibition of the activity of this enzyme is more effective in inhibiting tumor growth. In fact, direct or indirect pharmacological inhibitors that were able to reduce the activity of ChoK (and consequently the levels of tCho and PCho) did not reduce cell viability as long as ChoKα protein expression and PtdCho levels were not reduced in TNBC cells grown *in vitro* (75).

The potential of using PtdCho catabolic pathways as important cotargets for TNBC therapy is gaining relevance. A multi-targeting strategy such as simultaneous silencing of PLD1 and ChoKα in MDA-MB-231 cells increased apoptosis (detected by the TUNEL assay) as compared with individual treatments (47). Exposure of MDA-MB-231 cells to D609 which is an inhibitor of a PC-PLC resulted in 60–80% PC-PLC inhibition associated with tumor cell differentiation, detected by a progressive decrease of mesenchymal traits such as vimentin and N-cadherin expression, reduced galectin-3 and milk fat globule EGF-factor 8 levels, β-casein formation and decreased *in vitro* cell migration and invasion (32). These results, obtained from a single tumor model of TNBC, warrant further investigations on a large data set of human TNBCs that can be fully genotyped and metabolically characterized.

## Future Directions

Although evidence of specific metabolic alterations in TNBC is accruing, there is a clear need for extending preclinical investigations to a larger number of TNBC models. On the other hand, clinical investigations have to better elucidate the impact of the heterogeneous nature of TNBC lesions on the metabolic profiles and their changes in tumor progression. It may also prove relevant to assess the links between the tCho profile and molecular features such as EGFR overexpression, p53 status, and other specific biological TNBC characteristics. We hypothesize that the PtdCho cycle may represent a good focus point for personalized/precision medicine, offering markers that may be used as diagnosis tools for assessment of cancer prognosis and response to therapy.

The identification of a role for PtdCho metabolism in TNBC progression supports the view that some enzymes of this cycle may act as key regulators of molecular mechanisms leading to cancer onset, invasion, and metastasis, thus representing a new source of potential targets to counteract cancer growth and metastasis.

## Author Contributions

The manuscript was written by EI; revised by FP, MJC, RC, SC, FS, GC, and EI; read and approved by all coauthors.

## Conflict of Interest Statement

The authors declare that the research was conducted in the absence of any commercial or financial relationships that could be construed as a potential conflict of interest. The reviewer BK and handling editor declared their shared affiliation, and the handling editor states that the process nevertheless met the standards of a fair and objective review.
